# Clinical usefulness of the angle between left main coronary artery and left anterior descending coronary artery for the evaluation of obstructive coronary artery disease

**DOI:** 10.1371/journal.pone.0202249

**Published:** 2018-09-13

**Authors:** Seong Ho Moon, Joung Hun Byun, Jong Woo Kim, Sung Hwan Kim, Ki Nyun Kim, Jae Jun Jung, Dong Hoon Kang, Jun Ho Yang, Jun Young Choi, In Seok Jang, Hyun Oh Park, Chung Eun Lee, Jong Hwa Ahn

**Affiliations:** 1 Department of Thoracic and Cardiovascular Surgery, Changwon Hospital, Gyeongsang National University College of Medicine, Changwon, Republic of Korea; 2 Department of Thoracic and Cardiovascular Surgery, Jinju Hospital, Gyeongsang National University College of Medicine,Jinju, Republic of Korea; 3 Department of Cardiology, Gyeongsang National University College of Medicine,Changwon, Republic of Korea; Ziekenhuisgroep Twente, NETHERLANDS

## Abstract

**Background:**

A wider angle between the left anterior descending coronary artery (LAD) and left circumflex coronary artery (LCX) has been suggested to induce plaque formation in the arterial system via changes in shear stress. However, the relationship between the left main coronary artery (LM)-LAD angle and LAD stenosis has not been investigated. Therefore, we aimed to evaluate the associations between the LM-LAD and LAD-LCX angles and LAD stenosis.

**Methods:**

Coronary computed tomography angiographies (CTAs) of 201 patients with suspected coronary artery disease were analyzed. Angle measurements were performed twice by experts using CTA images, and the values were averaged. The patients were divided into two groups, based on the presence of significant LAD stenosis (luminal diameter narrowing ≥50%) on CTA.

**Results:**

The mean LM-LAD and LAD-LCX angles were 37.46° and 63.04°, respectively. The LM-LAD and LAD-LCX angles of the group with significant LAD stenosis were significantly wider than that of the group with nonsignificant LAD stenosis (P<0.001; P = 0.020, respectively). In a multivariate analysis, an LAD-LCX angle greater than 60° showed a trend toward predicting significant LAD stenosis (HR, 3.14; 95% CI: 0.96–1026; P = 0.058). In contrast, an LM-LAD angle greater than 40° was a significant predictor of significant LAD stenosis (HR, 12.2; 95% CI: 2.60–56.52; P = 0.001).

**Conclusions:**

The results of the present study may suggest that a wider LM-LAD angle could be used to identify patients at higher risk for coronary artery disease (CAD). Thus, close follow–up and preventive management of other risk factors may be needed in such cases.

## Introduction

Clinical concern for the coronary bifurcation angles, including the angles between the left main coronary artery (LM) and the left anterior descending coronary artery (LAD) and between the LAD and the left circumflex coronary artery (LCX), has increased, as hemodynamic changes resulting from variations in the shear stress according to the coronary bifurcation angle have an effect on plaque initiation in the arterial system.[1]. In general, low shear stress is considered atherogenic [2–5]. The majority of previous studies have reported that a wider LAD-LCX angle induces plaque formation in the arterial system [6]. However, to our knowledge, the relationship between the LM-LAD angle and LAD stenosis has not been investigated. Therefore, the aim of the present study was to evaluate the associations between the LM-LAD and LAD-LCX angles and LAD stenosis in patients with suspected CAD.

## Materials and methods

This retrospective study was approved by the Institutional Ethical Committee of Changwon Hospital, Gyeongsang National University School of Medicine (Approval number GNUCH 2017-11-004-002) and the requirement to obtain informed consent was waived due to the retrospective nature of the report.

All data were fully anonymized before we accessed them.

**Fig 1:**
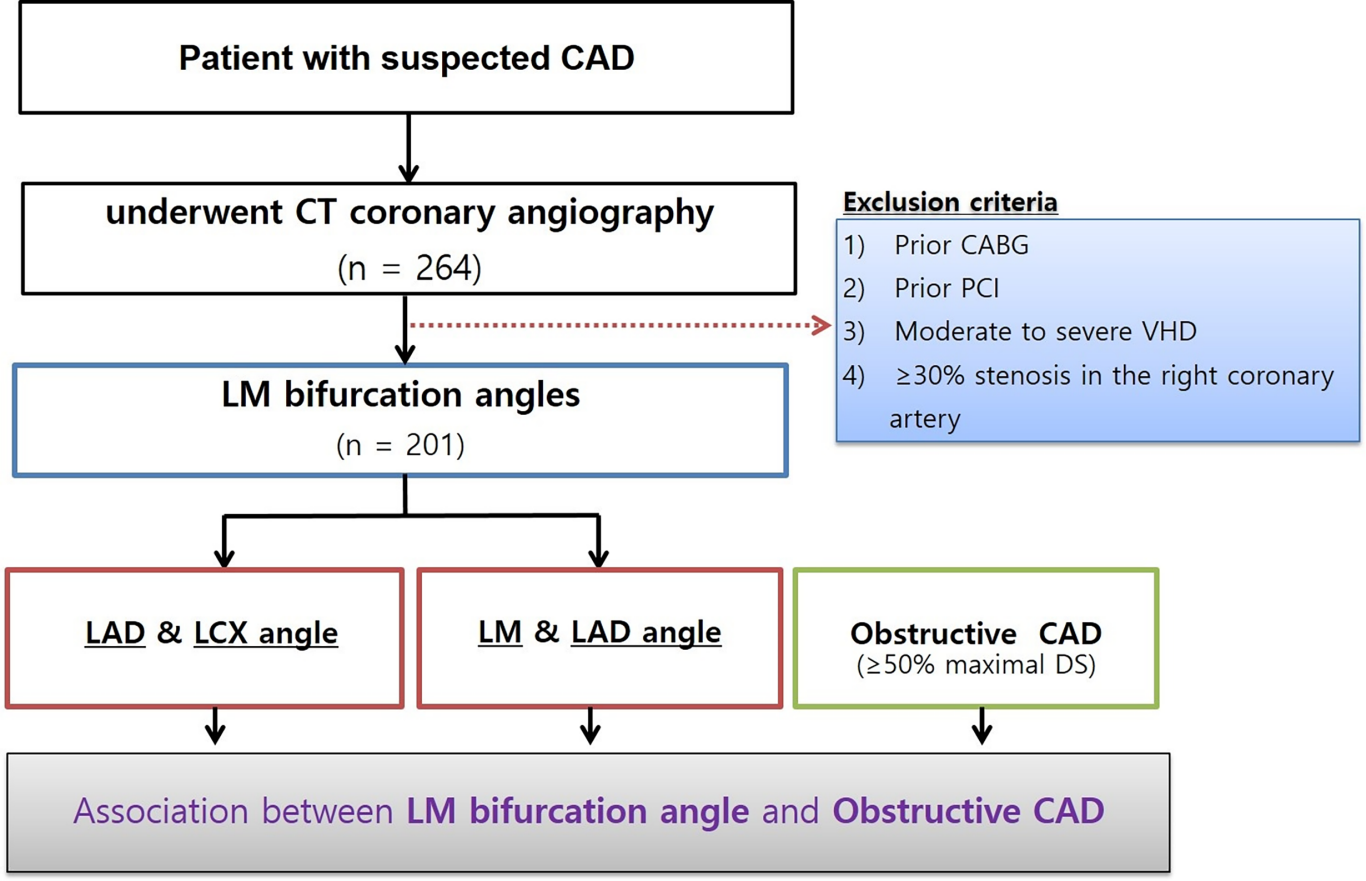
Study flow chart, including exclusion criteria.

**Fig 2:**
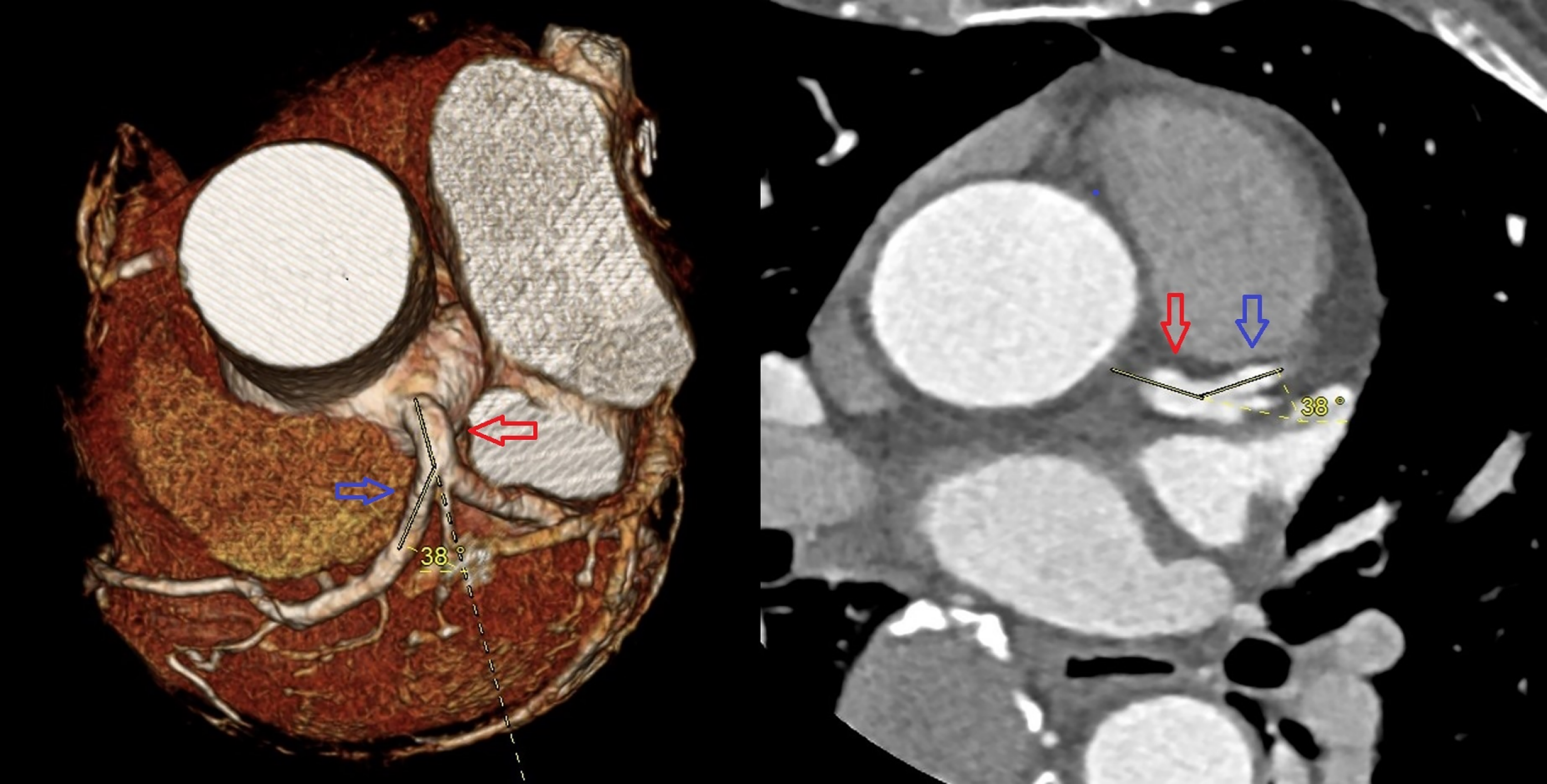
A representative image of angle measurement on computed tomography angiography. The angle between the left main coronary artery (red arrow) and the left anterior descending coronary artery (blue arrow) is 38°.

CAD, coronary artery disease; CT, computed tomography; CABG, coronary artery bypass grafting, PCI, percutaneous coronary intervention; VHD, valvular heart disease; DS, diameter stenosis; LM, left main coronary artery; LAD, left anterior descending coronary artery; LCX, left circumflex coronary artery.

The data from 264 consecutive patients with suspected CAD who underwent computed tomography angiography (CTA) due to angina symptoms at our hospital in 2016 were reviewed. Among these, 63 patients were excluded and the remaining 201 patients were enrolled. The patient selection flowchart, with the exclusion criteria, is shown in Fig 1. The LM-LAD and LAD-LCX angles were measured using three-dimensional volume rendering images and two-dimensional axial images (Fig 2). All measurements were performed by experienced cardiologists and cardiothoracic surgeons. All angles were measured twice, and average values were analyzed, minimizing the influence of inter-observer disagreements. The patients were separated into two groups, depending on the presence of significant LAD stenosis (luminal diameter narrowing ≥50%) on CTA, as interpreted by radiologists.

### CTA protocol and image analysis

CTA was performed using a Brilliance 64 multi-detector scanner (Philips Healthcare, Best, the Netherlands). Patients with a heart rate >80 beats/min received oral propranolol, given in 40-mg increments (up to 120 mg). Sublingual nitroglycerin was administered 1 min before scanning. A bolus of 80–100 mL of nonionic contrast medium was injected intravenously, at a flow rate of 5 mL/s, followed by an injection of saline (50 mL at 5 mL/s). After the contrast injection, a retrospective electrocardiography (ECG)-gated spiral scan was performed, covering the region immediately beneath the aortic arch to the apex of the left ventricle during an inspiratory breath hold of 10–20 ms, depending on the particular scanner. The scanning parameters were as follows: gantry rotation time, 330–420 ms; dose modulation (ECG pulsing), 750–850 mA and 120 kV; and slice thickness, 0.75 mm. For all scanners, a multi-segment algorithm was used to reconstruct overlapping images, typically covering 75% of the cardiac cycle.

### Statistical analysis

The distribution of continuous variables was evaluated for normality using the Kolmogorov-Smirnov test. Continuous variables with normal distribution are presented as means ± standard deviation, and were compared between groups using the Student’s t-test. Chi-square or Fisher’s exact tests were used to evaluate group differences in categorical variables. Receiver operating characteristic (ROC) curves were constructed to further analyze the usefulness of the coronary bifurcation angles in predicting significant LAD stenosis. In addition, univariate and multivariate binary logistic regression analyses were performed to determine the predictors of LAD stenosis and the hazard ratios (HRs) and 95% confidence intervals (CIs) were calculated. All statistical analyses were performed using SPSS for Windows, version 21 (IBM Corporation, Chicago, IL). A two-tailed P-value <0.05 was considered significant.

## Result

Baseline patient characteristics are shown in Table 1. The two groups significantly differed in age (P = 0.043), with no other significant differences in the baseline characteristics. Among all patients, the mean LM-LAD and LAD-LCX angles were 37.46° and 63.04°, respectively. The LM-LAD and LAD-LCX angles were significantly wider in the group with significant LAD stenosis (≥50%) compared to that in the group with nonsignificant LAD stenosis (<50%) (P<0.001 and P = 0.020, respectively) (Table 2). 17 out of 18 patients with significant LAD stenosis had LAD proximal or mid lesion.

**Table 1:** Baseline characteristics according to the presence of significant LAD stenosis (total n = 201).

	LAD stenosis ≥ 50% (n = 18)	LAD stenosis < 50% (n = 183)	P-value
Age (year)	64.4 ± 9.6	57.9 ± 13.2	0.043
Male (%)	11 (61.1)	99 (54.1)	0.568
Body mass index (kg/m^2^)	24.8 ± 3.1	24.4 ± 3.6	0.684
Diabetes (%)	4 (22.2)	23 (12.6)	0.252
Current smoker (%)	3 (16.7)	38 (20.8)	0.681
Dyslipidemia (%)	1 (5.6)	47 (25.7)	0.056
Hypertension (%)	8 (44.4)	78 (42.6)	0.882

Data presented as numbers (%) or means **±** standard deviation. LAD, left anterior descending artery.

**Table 2:** Coronary angles according to the presence of significant LAD stenosis.

Angle	LAD stenosis ≥50%	LAD stenosis < 50%	P- value
LM-LAD (°)	49.4 ± 9.0	36.2 ± 9.7	<0.001
LAD-LCX (°)	72.9 ± 23.3	62.1 ± 18.2	0.020

Data presented as means ± standard deviation. LM, left main coronary artery; LAD, left anterior descending artery; LCX, left circumflex artery.

**Fig 3:**
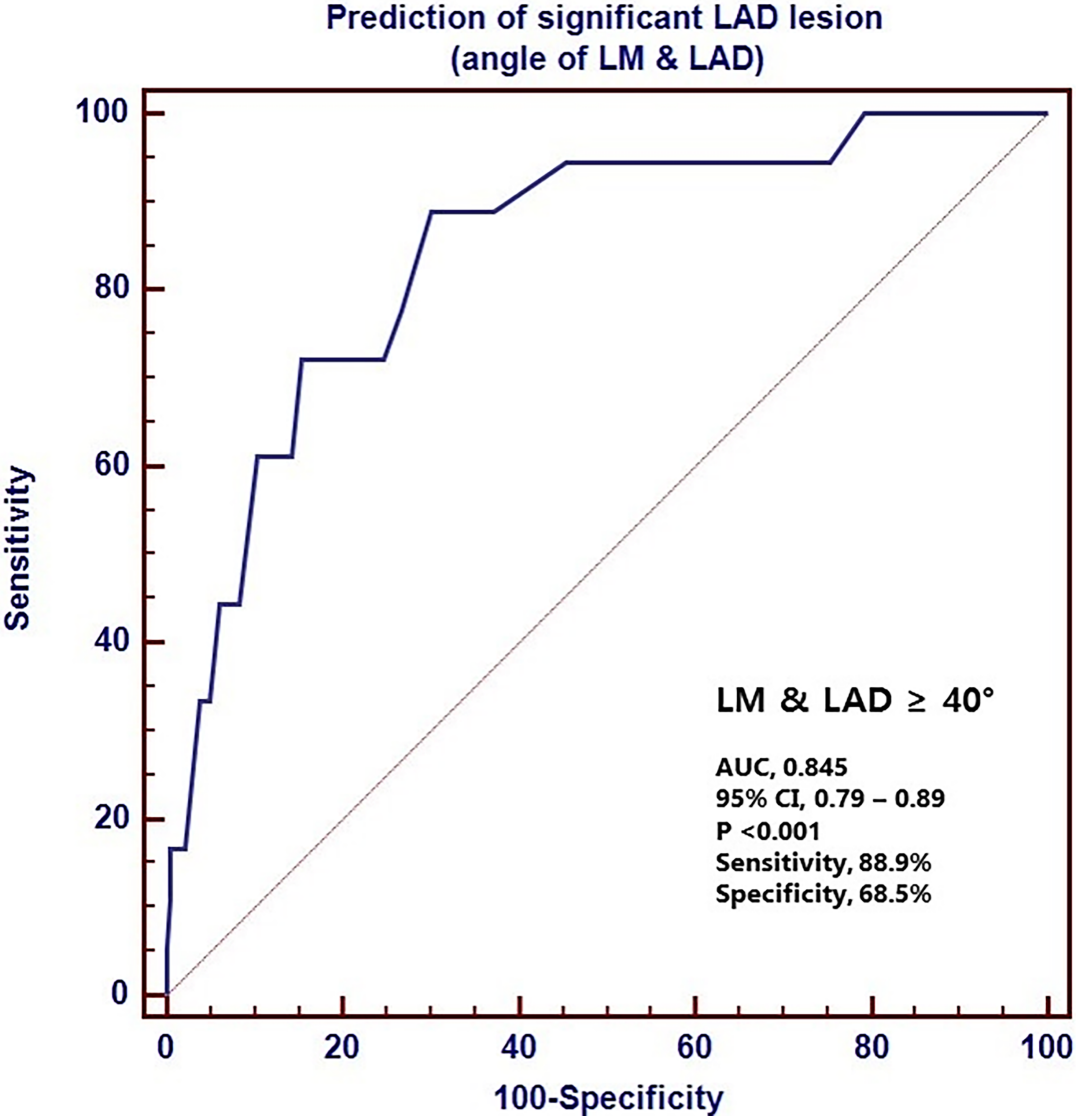
The receiver operating characteristics (ROC) curve for the LM-LAD angle in the prediction of LAD stenosis. The area under the curve (AUC) is 0.845. LM, left main coronary artery; LAD, left anterior descending coronary artery; CI, confidence interval.

**Fig 4:**
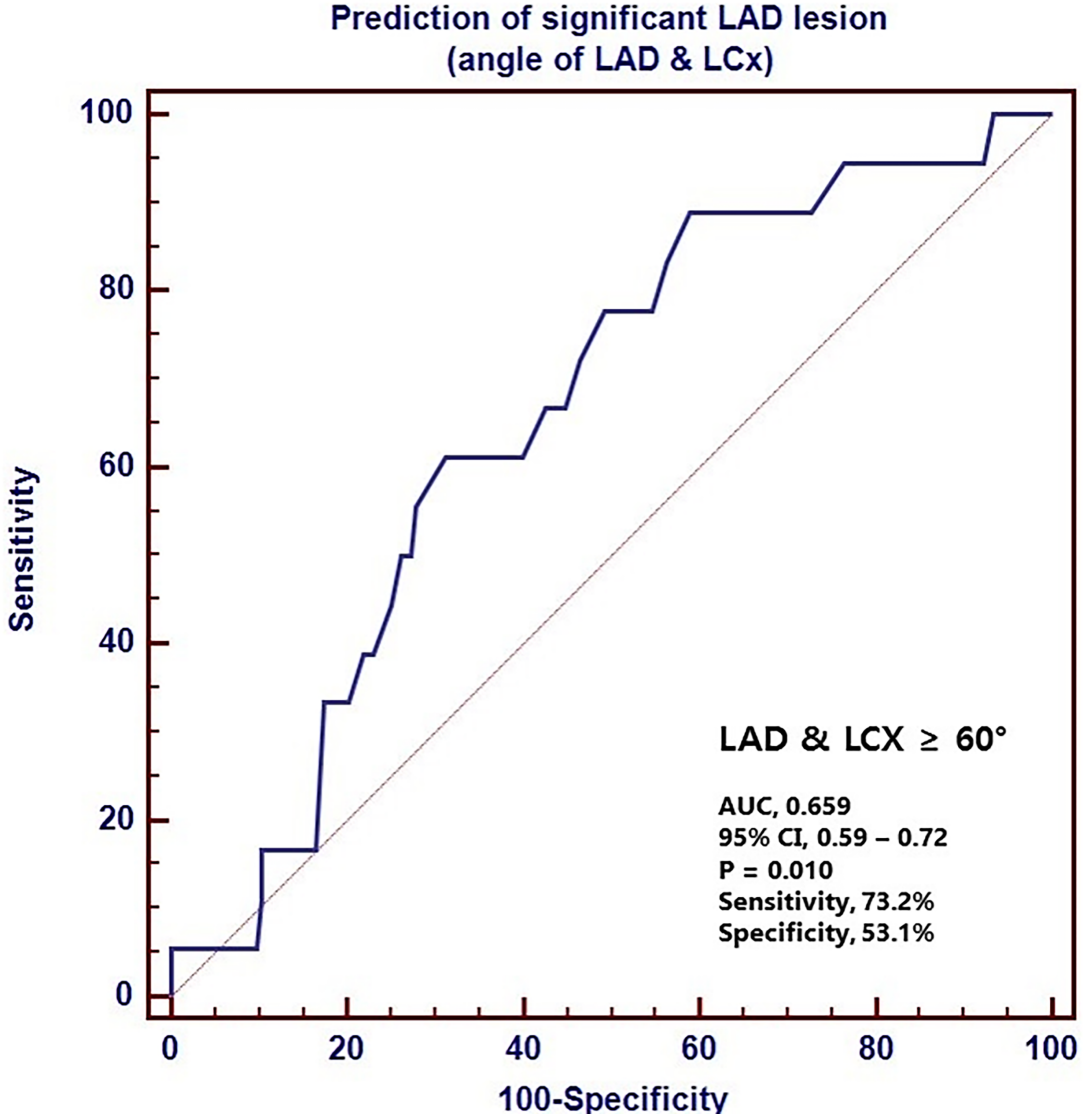
The receiver operating characteristics (ROC) curve for the LAD-LCx angle in the prediction of LAD stenosis. The area under the curve (AUC) is 0.659. LAD, left anterior descending coronary artery; LCX, left circumflex coronary artery; CI, confidence interval.

The ROC curves for the LM-LAD and LAD-LCX angles had an area under the curve of 0.845 and 0.659, respectively (Figs 3 and 4). The cut-off values of 40° and 60° for the LM-LAD and LAD-LCX angles, respectively, had sensitivities of 88.9 and 73.2, respectively, and specificities of 68.5 and 53.1, respectively. In univariate analyses, LM-LAD and LAD-LCX angles greater than 40° and 60°, respectively, were significant predictors of significant LAD stenosis (HR, 15.24; 95% CI: 3.40–68.38; P<0.001; and HR, 3.62; 95% CI: 1.15–11.40; P = 0.028, respectively). However, in the multivariate analysis, an LAD-LCX angle >60° only showed a trend toward predicting significant LAD stenosis (HR, 3.14; 95% CI: 0.96–1026; P = 0.058). In contrast, an LM-LAD angle >40° was a significant independent predictor of significant LAD stenosis (HR, 12.2; 95% CI: 2.60–56.52; P = 0.001) (Table 3).

**Table 3:** Results of the univariate and multivariate logistic regression analyses for predictors of significant LAD stenosis.

			Analysis			
	LAD stenosis ≥50%	LAD stenosis <50%	Univariate OR (95% CI)	P	Multivariate OR (95% CI)	P
LM-LAD > 40°	16/79 (20.3%)	2/122 (1.6%)	15.24 (3.40–68.38)	<0.001	12.12 (2.60–56.52)	0.001
LAD-LCX >60°	14/104 (13.5%)	4/97 (4.1%)	3.62 (1.15–11.40)	0.028	3.14 (0.96–10.26)	0.058

CI, confidence interval; OR, odds ratio; LM, left main coronary artery; LAD, left anterior descending artery; LCX, left circumflex artery.

Of the 60 patients with LAD lesions, 49 (70%) patients had LM-LAD angle greater than 40°. Of these, 47 (96%) patients had proximal or mid LAD lesion.

## Discussion

Atherosclerosis is associated with systemic inflammation of the arterial system via intimal lesion formation 1. Atherosclerotic plaque rupture can lead to cerebral and cardiovascular events. In general, low shear stress is considered atherogenic [2–5]. A study of LM bifurcations reported that LM-LAD tortuosity appeared to be a predictor of low shear stress, whereas LM-LCX tortuosity was not predictor of shear stress [6].

Wall shear stress (τ) was then calculated using the equation:τ = 4 ηQ/*πr*^3^

Where η is the apparent blood viscosity (0.035 Poise) and Q is the blood flow rate through the vessel [7]. r is the radius of vessels. The shear stress increases when blood flow increases or blood vessel diameter decreases. The shear stress decreases as the blood flow decreases.

A porcine study reported that low shear stress initiated plaque onset and the occurrence of vulnerable plaques [8]. Furthermore, vascular regions exposed to low shear stress show enhanced endothelial activation, characterized by reduced nitric oxide (NO) production and enhanced oxidant stress and proinflammatory activation [9].

A wider left coronary bifurcation angle has been reported to form a region of low shear stress in bifurcating regions [10, 11]. Several studies have evaluated relationships between left coronary bifurcation angles and the development of CAD [12–16], with most demonstrating an association between the LAD-LCX angle and CAD. However, the ratio of the LM-LAD and LM-LCX angles constituting the LAD-LCX angle may differ among patients, even if the LAD-LCX angle is the same. When the contribution of the LM-LAD angle is large, a region of low shear stress in the bifurcating region (LM-LAD) may form. For instance, a lower shear stress may act on the LAD with LM-LAD and LM-LCX angles of 60° and 40°, respectively, compared to that with LM-LAD and LM-LCX angles of 50° each. In other words, even if the same LAD-LCX angle is seen in each patient, the shear stress actually acting on the LAD will depend on the LM-LAD angle. As shown in the above example, the LM-LAD angle may be small or large even if the LAD-LCX angle is actually the same, and the low shear stress may be applied when the LM-LAD angle is large.

The results of the present study suggest that the LM-LAD angle is a more accurate predictor of significant LAD stenosis than the LAD-LCX angle. In the multivariate analysis, the association between significant LAD stenosis and the LAD-LCX angle failed to reach significance, showing only a trend (P = 0.058). Thus, the shear stress of the LAD may be affected by the LM-LAD angle, rather than the LAD-LCX angle itself.

In the present study, the LM-LAD angle cut-off value for the prediction of significant LAD stenosis was 40°. Similarly, Konishi T et al. [17]reported that the LM-LAD angle cut-off value for the prediction of restenosis after stenting of the proximal LAD was 34°. This previous study also showed that the low shear stress of the wider angle in this region is atherogenic, consistent with our explanation of the relationship between the LM-LAD angle and significant LAD stenosis. Previous studies have reported a cut-off value of 80° for the LAD-LCX angle in predicting the presence of CAD [18, 19]. In contrast, the present study found a cut-off value of 60° for LAD-LCX angle in predicting the presence of CAD. As the sample size was relatively small in the present study, a statistical bias may have influenced the results, and further studies are required.

## Limitations

The sample size of this retrospective study, performed at a single center, was small. In particular, the number of patients with significant LAD stenosis was small. Thus, a larger, multi-center, prospective study is needed. As intravascular ultrasonography or coronary angiography was not performed in most of the patients, the angle was measured on multi-detector CT alone. However, the presence and severity of LAD stenosis on CT was measured by two or more radiologists, and angles were measured by two cardiovascular surgeons and cardiologists for each patient.

## Conclusion

The results of the present study may suggest that a wider LM-LAD angle could be used to identify patients at higher risk for CAD. Thus, close follow–up and preventive management of other risk factors may be needed in such cases.

## Supporting information

S1 File Dataset. (XLSX)

## Abbreviations

LM: left main coronary artery
LAD: left anterior descending coronary artery
LCX: left circumflex coronary artery
CTA: Coronary computed tomography angiography
CAD: coronary artery disease
